# Editorial: Advanced Sampling and Modeling in Molecular Simulations for Slow and Large-Scale Biomolecular Dynamics

**DOI:** 10.3389/fmolb.2021.795991

**Published:** 2021-11-12

**Authors:** Xiakun Chu, Yong Wang, Pengfei Tian, Wenfei Li, Davide Mercadante

**Affiliations:** ^1^ Department of Chemistry, State University of New York, Stony Brook, NY, United States; ^2^ College of Life Sciences, Shanghai Institute for Advanced Study, Institute of Quantitative Biology, Zhejiang University, Hangzhou, China; ^3^ Enzyme Research, Novozymes, Lyngby, Denmark; ^4^ National Laboratory of Solid State Microstructure, Department of Physics, Nanjing University, Nanjing, China; ^5^ Wenzhou Institute, University of Chinese Academy of Sciences, Wenzhou, China; ^6^ School of Chemical Sciences, The University of Auckland, Auckland, New Zealand

**Keywords:** molecular dynamics, enhanced sampling, coarse-grained model, conformational dynamics, free energy landscape

From conception, the impact of molecular dynamics (MD) simulations has grown dramatically (Karplus and McCammon, 2002), even though MD simulations are still affected by a critical timescale issue. Currently, timescales accessible to MD simulations are, on average, shorter than those of the investigated events, often resulting in insufficient sampling. Numerous efforts have been spent on accelerating MD simulations in order to ease the timescale problem. This Research Topic collects contributions focusing on developing and using advanced sampling techniques and modeling strategies to promote applications of MD simulations to study a diverse range of large-scale biomolecular systems.

Exhaustive sampling is especially important for intrinsically disordered proteins (IDPs), which show a more shallow and rugged energy landscape when compared to folded proteins (Papoian, 2008). Ding et al. presented case studies on two IDPs by an iterative screening-after-sampling strategy. In their study accelerated molecular dynamics was used to enhance the sampling of highly diverse conformational ensembles of IDPs, with Small-angle X-ray scattering (SAXS) to guide the sampling iteratively and obtain ensembles in good agreement with experimental data. Such integrative modeling might be more broadly useful for modeling IDPs ensembles. In general, the conformational dynamics of IDPs are strongly affected by the binding to other molecular partners or by aggregation. There are three excellent studies in this Research Topic, focusing on the conformational dynamics and aggregation properties of two Alzheimer’s disease-related IDPs: the A*β*42 peptide and the tau protein (Selkoe and Hardy, 2016). In the work by Xie and Guo, replica exchange with solute tempering (REST) (Liu et al., 2005) has been adopted to sample the binding of the intrinsically disordered A*β*42 peptide to the Human serum albumin (HSA), elucidating the molecular mechanism of amyloid inhibition by HSA. Their results suggest that A*β*42 binds to multiple sites on HSA, which shifts the conformational propensity of the peptide towards a more disordered state altering its aggregation propensity altogether. The reward behind the quest to mechanistically characterize fibrillar nucleation in proteopathies is enormous as it would suggest effective strategies for therapeutic approaches to neurodegeneration. Ma et al. employed coarse-grained simulations to energetically define the fibril growth of the A*β*-peptide: discovering the binding site of new filaments to the protofibril and a downhill mechanism of filament addition. Together with the identification of an emerging mechanical property of the A*β*-peptide protofibril, their research adds valuable insights to our understanding of the nucleation of A*β*-peptide fibrils for the development of strategies to pharmaceutically tackle fibril growth. McCarty and co-workers performed non-equilibrium steered MD simulations to investigate the structural changes of the tau paired-helical filament (PHF) and straight filament (SF) under mechanical force. In particular, the authors identified weak spots of interchain interactions additionally providing the dissociation pathway of a single tau peptide from the protofibril, through metadynamics simulations (Laio and Parrinello, 2002). In addition, the free energy profile for tau dissociation was obtained by umbrella sampling simulations.

One useful strategy to overcome sampling limitations is to introduce coarse-graining into biomolecular models. MARTINI is among the most widely used coarse-grained (CG) models for biomolecules (Monticelli et al., 2008). Mahmood et al. introduced a simple cutoff scheme to improve the definition of native contacts in the structure-based protein model of MARTINI (G $\bar{o}$ -MARTINI). By tuning the interaction strengths and cutoff distances, the MARTINI CG simulations can well reproduce the structural fluctuations from atomistic simulations for the membrane proteins investigated in the published study. The refined model has been successfully used to simulate the key steps leading to the assembly of the F-BAR protein involved in membrane remodeling. Using the CG UNited-RESidue (UNRES) model (Liwo et al., 1997), Stevens and He studied the large-scale conformational changes within the multidomain scaffolding protein PICK1. Considering the large size of PICK1, associated with extensive conformational flexibility, brute-force atomistic simulations on this system would inevitably lead to insufficient sampling. The physics-based CG model allowed the authors to reliably characterize the detailed interactions at the residual level and eventually uncover the forces driving the association of PICK1 subdomains. CG modeling has also been suggested to study and understand chromosomal organization and dynamics (Lin et al., 2021). However, determining the potential energy function suitably reproducing the behaviour of chromosomal and multi-chromosomal systems has been historically challenging. Zhang and Huang introduced a “bottom-up” approach, aiming to derive the CG potential from all-atom MD simulations. They studied dynamics and interactions in two-nucleosome systems, providing useful information to determine functional forms and parameters to sample the interaction between nucleosomes.

Coarse-graining the MD simulation trajectories is another useful strategy to reduce system complexity, while elucidating molecular dynamics. In addition to the sampling problem, extracting key structural and dynamic features from high-dimensional MD trajectories is a challenge in biomolecular modeling. Such a task relies on an appropriate definition of feature space, within which the metastable states involved in biologically relevant conformational dynamics can be identified *via* clustering and dimensionality reduction analyses. The humanly understandable thermodynamic and kinetic information can then be reconstructed based on Markov State Models (MSM) (Chodera and Noé, 2014; Lane et al., 2011). In the work by Wang et al., an effective energy rescaling space trajectory mapping method has been developed to detect metastable states and construct kinetic transition networks. In their study the authors are able to successfully describe the major metastable states and the interstate transition kinetics involved in the folding of a dodecapeptide. MSM can be combined with enhanced sampling methods to further improve the performance of structural and dynamic characterizations as done by Fernández-Quintero et al. In this work, the authors demonstrated the correlation between the rigidification of the CDR-H3 loops of antibody fragments and the enhanced antigen specificity in different stages of affinity maturation by using metadynamics simulations in combination with MD simulations and MSM analyses.

This Research Topic also includes two binding case studies. Do et al. employed all-atom MD simulations with the aid of an enhanced sampling method called Gaussian-accelerated molecular dynamics (GaMD) (Wang et al., 2021) to determine the pathways and binding mechanism of caffeine to the human adenosine A2A receptor. By adding a harmonic boost potential, GaMD simulations allowed to capture the spontaneous ligand binding and release in the μs time scale through smoothening the potential energy surface so as to reduce the energy barriers for slow conformational changes. This work provided a good example on how to implement enhanced sampling methods to study a protein-ligand binding mechanism. In investigating the binding between nanoparticles and the clathrin-associated protein adaptin-2 (AP2), Zhu et al. applied molecular modeling and simulations to understand the impact of nanoparticle morphology on binding specificity. They found that binding specificity is majorly dictated by electrostatic interactions as well as nanoparticle morphology. They also observed that nanoparticle binding significantly induces conformational changes in AP2. Overall, the authors provided a microscopic explanation for cargo recognition in clathrin-mediated endocytosis and possible mechanisms to design high-efficiency nano-biomaterials.

As an essential element in MD simulations, the adopted force field is key to affect the precision of the simulation results. Wang and Li developed and tested force field parameters for some noncanonical amino acids (NAAs). NAAs have been widely applied in protein engineering, virus vaccine development, and medical therapeutics due to their strong site specificity, without the need to introduce significant perturbations to a protein structure. Based on quantum mechanics (QM) calculations and experimental data as a benchmark, the authors determined force field parameters for phenylalanine and tyrosine derivatives showing that the newly identified parameters well describe protein-ligand interactions with NAAs as substrates. Finally, to aid structural modeling useful for MD simulations, Xian et al. developed a structure manipulation (StructureMan) tool that proved to be comprehensive and efficient when studying interactions in large biomolecular systems.

Overall, we believe that this Research Topic provides a well-rounded picture of the latest state-of-the-art developments useful to overcome historical limitations in modeling and sampling of large biomolecular systems and slow processes using classical molecular dynamics simulations.
